# Genomic Surveillance for SARS-CoV-2 Variants: Circulation of Omicron Lineages — United States, January 2022–May 2023

**DOI:** 10.15585/mmwr.mm7224a2

**Published:** 2023-06-16

**Authors:** Kevin C. Ma, Philip Shirk, Anastasia S. Lambrou, Norman Hassell, Xiao-yu Zheng, Amanda B. Payne, Akilah R. Ali, Dhwani Batra, Jason Caravas, Reina Chau, Peter W. Cook, Dakota Howard, Nicholas A. Kovacs, Kristine A. Lacek, Justin S. Lee, Duncan R. MacCannell, Lakshmi Malapati, Sandra Mathew, Neha Mittal, Roopa R. Nagilla, Rishika Parikh, Prabasaj Paul, Benjamin L. Rambo-Martin, Samuel S. Shepard, Mili Sheth, David E. Wentworth, Amber Winn, Aron J. Hall, Benjamin J. Silk, Natalie Thornburg, Rebecca Kondor, Heather M. Scobie, Clinton R. Paden

**Affiliations:** ^1^National Center for Immunization and Respiratory Diseases, CDC; ^2^Epidemic Intelligence Service, CDC; ^3^National Center for Emerging and Zoonotic Infectious Diseases, CDC; ^4^General Dynamics Information Technology, Inc., Atlanta, Georgia; ^5^Tanaq Support Services, LLC, St. George Tanaq Corporation, Anchorage, Alaska; ^6^Eagle Global Scientific, LLC, Atlanta, Georgia; ^7^Goldbelt C6, Chesapeake, Virginia.

CDC has used national genomic surveillance since December 2020 to monitor SARS-CoV-2 variants that have emerged throughout the COVID-19 pandemic, including the Omicron variant. This report summarizes U.S. trends in variant proportions from national genomic surveillance during January 2022–May 2023. During this period, the Omicron variant remained predominant, with various descendant lineages reaching national predominance (>50% prevalence). During the first half of 2022, BA.1.1 reached predominance by the week ending January 8, 2022, followed by BA.2 (March 26), BA.2.12.1 (May 14), and BA.5 (July 2); the predominance of each variant coincided with surges in COVID-19 cases. The latter half of 2022 was characterized by the circulation of sublineages of BA.2, BA.4, and BA.5 (e.g., BQ.1 and BQ.1.1), some of which independently acquired similar spike protein substitutions associated with immune evasion. By the end of January 2023, XBB.1.5 became predominant. As of May 13, 2023, the most common circulating lineages were XBB.1.5 (61.5%), XBB.1.9.1 (10.0%), and XBB.1.16 (9.4%); XBB.1.16 and XBB.1.16.1 (2.4%), containing the K478R substitution, and XBB.2.3 (3.2%), containing the P521S substitution, had the fastest doubling times at that point. Analytic methods for estimating variant proportions have been updated as the availability of sequencing specimens has declined. The continued evolution of Omicron lineages highlights the importance of genomic surveillance to monitor emerging variants and help guide vaccine development and use of therapeutics.

CDC’s national genomic surveillance system integrates SARS-CoV-2 sequences from three sources: 1) the National SARS-CoV-2 Strain Surveillance (NS3) program,^†^ 2) CDC-contracted commercial laboratories, and 3) public sequence data repositories, including the Global Initiative on Sharing All Influenza Data (GISAID) repository and National Center for Biotechnology Information (NCBI) GenBank.^§^ Variant proportions generated by genomic surveillance are regularly updated on CDC’s COVID Data Tracker and guide public health measures to address COVID-19^¶^ (*1*,*2*).

Weekly SARS-CoV-2 consensus sequences** from the NS3 program, commercial laboratories, and data repositories were quality-filtered,^††^ deduplicated, and assigned Pango lineages (*3*). During January 2022–May 2023, the median interval from specimen collection to data availability was 16 days. Weekly variant proportions were estimated at the national and U.S. Department of Health and Human Services (HHS) regional levels^§§^ by specimen collection date for the 11 weeks before the most recent 3 weeks; lineages were included if they constituted ≥1% (unweighted) of sequences nationally and contained spike protein substitutions of potential therapeutic relevance. To estimate variant proportions for the most recent 3 weeks, nowcasts were generated using multinomial regression fit on the previous 21 weeks of data.^¶¶^ All methods included weighting to account for the complex survey design and adjust for potential sampling biases.*** Nowcasts were conducted for any lineages with ≥0.5% prevalence beginning October 11, 2022,^†††^ to improve accuracy by accounting for differential growth rates of grouped sublineages. Weekly numbers of COVID-19 cases attributable to variants were estimated by multiplying counts of positive nucleic acid amplification tests from COVID-19 electronic laboratory reporting (CELR) with variant proportions. Doubling times for proportions of specific lineages were estimated from the coefficients of the multinomial nowcasting model.^§§§^ Methodologic changes following the public health emergency expiration (*4*) were summarized. Biweekly estimates using the updated model were compared with weekly estimates from the previous model to assess consistency. Data were current as of June 1, 2023. This activity was reviewed by CDC and conducted consistent with applicable federal law and CDC policy.^¶¶¶^

During January 2, 2022–May 13, 2023, a total of 1,697,197 SARS-CoV-2 surveillance sequences from 56 U.S. jurisdictions**** were generated by or reported to CDC from NS3 (1%), commercial laboratories (60%), and repositories (38%); the percentage of sequences from repositories represented an increase from 10% during June 2021–January 2022 (*1*). The weekly number of sequenced specimens decreased from approximately 65,000 collected in January 2022 to approximately 4,400 in April 2023, as the number of COVID-19 cases declined (Supplementary Figure 1, https://stacks.cdc.gov/view/cdc/129515).

Omicron remained predominant during January 2, 2022–May 13, 2023, with various descendent lineages emerging and becoming predominant nationwide. The BA.1.1 lineage reached predominance by the week ending January 8, 2022, followed by BA.2 by March 26, BA.2.12.1 by May 14, and BA.5 by July 2 (Figure 1). The prevalence of these lineages peaked at 75.7% (95% CI = 73.8%–77.5%) for BA.1.1 by the week ending February 19, 2022; 73.4% (95% CI = 69.6%–77.0%) for BA.2 by April 16; 62.4% (95% CI = 60.7%–64.0%) for BA.2.12.1 by May 28; and 86.2% (95% CI = 85.2%–87.2%) for BA.5 by August 20. Circulation of these lineages coincided with surges in COVID-19 cases (Figure 1) (Supplementary Figure 2, https://stacks.cdc.gov/view/cdc/129516).

**FIGURE 1:**
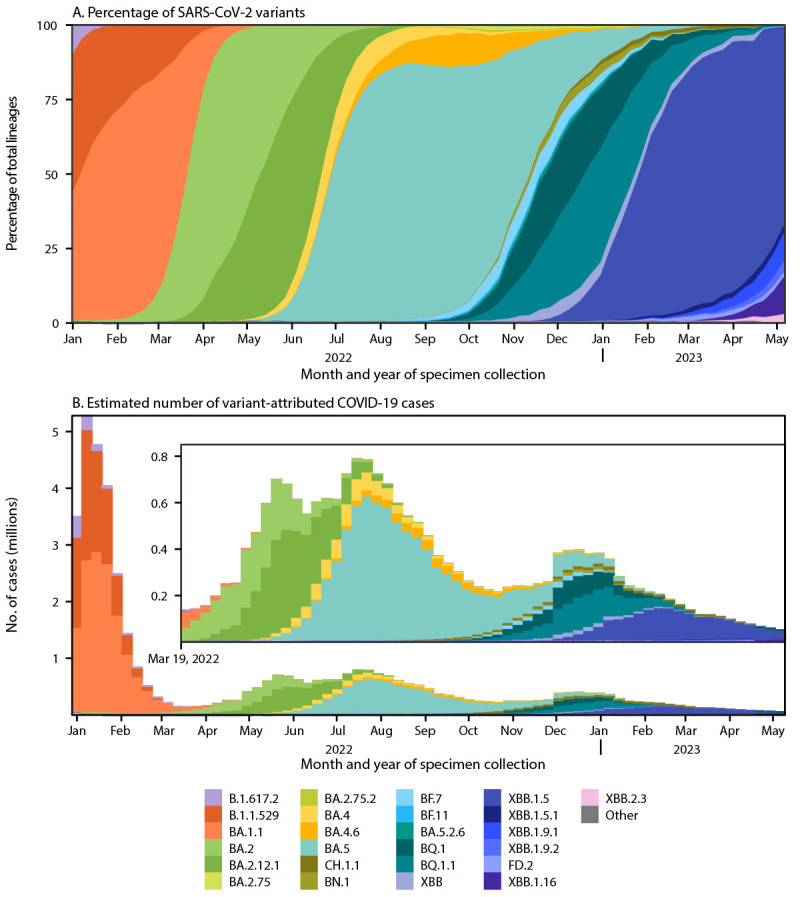
National estimates of weekly proportions* of SARS-CoV-2 variants^†^ (A) and estimated number of variant-attributed cases^§^ (B) — United States, January 2, 2022–May 13, 2023 **Abbreviations**: CELR = COVID-19 electronic laboratory reporting; NS3 = National SARS-CoV-2 Strain Surveillance Program. * Sequences are reported to CDC through NS3, contract laboratories, public health laboratories, and other U.S. institutions. Variant proportion estimation methods use a complex survey design and statistical weights to account for the probability that a specimen is sequenced. https://covid.cdc.gov/covid-data-tracker/#variant-proportions ^†^ Lineages reaching a prevalence of ≥1% with spike protein substitutions of potential therapeutic relevance and separated out on the COVID Data Tracker website. ^§^ Estimated numbers of COVID-19 cases attributable to variants were calculated by multiplying weekly numbers of reported positive nucleic acid amplification tests from CELR with estimated variant proportions.

During the latter half of 2022, multiple Omicron descendants of BA.2, BA.4, and BA.5,^††††^ including BA.2.75, BA.4.6, BF.7, BQ.1, BQ.1.1, BA.5.2.6, BN.1, BF.11, and CH.1.1 accounted for >1% of circulating variants at different points (Figure 1). Several of these lineages independently acquired spike receptor binding domain (RBD) substitutions, including R346T, K444T, N460K, and F486S/P (Table). None attained predominance individually; however, BQ.1 (which includes K444T and N460K) and BQ.1.1 (which also includes R346T) reached a combined peak prevalence of 59.3% by December 24, 2022 (BQ.1, 22.1%; BQ.1.1, 37.2%), coinciding with a winter surge in cases (Figure 1) (Supplementary Figure 2, https://stacks.cdc.gov/view/cdc/129516).

**TABLE:** Predominant amino acid substitutions* in the receptor binding domain (residues 333–527) of the spike protein among Omicron lineages with ≥1% prevalence^†^ relative to BA.4/BA.5 — United States, January 2, 2022–May 13, 2023

Lineage (partially expanded name)	Date added to CDT	Spike RBD (residues 333–527) amino acid substitutions																	
		339^§^	346^§,¶^	368	371	376	405	408	444^§,¶^	445^¶^	446^§,¶^	452^§,¶^	460^§,¶^	478	486^§,¶^	490^§,¶^	493^§^	496	521
**BA.4/BA.5 reference sequence**	**Jun 4, 2022**	**D**	**R**	**L**	**F**	**A**	**N**	**S**	**K**	**V**	**G**	**R**	**N**	**K**	**V**	**F**	**Q**	**G**	**P**
BA.4.6	Jul 30, 2022	—**	T	—	—	—	—	—	—	—	—	—	—	—	—	—	—	—	—
BA.5.2.6	Oct 29, 2022	—	T	—	—	—	—	—	—	—	—	—	—	—	—	—	—	—	—
BF.7 (BA.5.2.1.7)	Sep 17, 2022	—	T	—	—	—	—	—	—	—	—	—	—	—	—	—	—	—	—
BF.11 (BA.5.2.1.11)	Nov 19, 2022	—	T	—	—	—	—	—	—	—	—	—	—	—	—	—	—	—	—
BQ.1 (BA.5.3.1.1.1.1.1)	Oct 15, 2022	—	—	—	—	—	—	—	T	—	—	—	K	—	—	—	—	—	—
BQ.1.1 (BA.5.3.1.1.1.1.1.1)	Oct 15, 2022	—	T	—	—	—	—	—	T	—	—	—	K	—	—	—	—	—	—
BA.1.1	Feb 12, 2022	—	K	—	L	T	D	R	—	—	S	L	—	—	F	—	R	S	—
BA.2	Feb 5, 2022	—	—	—	—	—	—	—	—	—	—	L	—	—	F	—	R	—	—
BA.2.12.1	Apr 16, 2022	—	—	—	—	—	—	—	—	—	—	Q	—	—	F	—	R	—	—
BA.2.75	Sep 17, 2022	H	—	—	—	—	—	—	—	—	S	L	K	—	F	—	—	—	—
BN.1 (BA.2.75.5.1)	Nov 12, 2022	H	T	—	—	—	—	—	—	—	S	L	K	—	F	S	—	—	—
CH.1.1 (BA.2.75.3.4.1.1.1.1)	Jan 28, 2023	H	T	—	—	—	—	—	T	—	S	—	K	—	S	—	—	—	—
XBB/XBB.1	Nov 26, 2022	H	T	I	—	—	—	—	—	P	S	L	K	—	S	S	—	—	—
XBB.1.5	Dec 31, 2022	H	T	I	—	—	—	—	—	P	S	L	K	—	P	S	—	—	—
XBB.1.5.1	Mar 11, 2023	H	T	I	—	—	—	—	—	P	S	L	K	—	P	S	—	—	—
FD.2 (XBB.1.5.15.2)	Apr 15, 2023	H	T	I	—	—	—	—	—	P	S	L	K	—	P	S	—	—	—
XBB.1.9.1	Apr 1, 2023	H	T	I	—	—	—	—	—	P	S	L	K	—	P	S	—	—	—
XBB.1.9.2	Apr 15, 2023	H	T	I	—	—	—	—	—	P	S	L	K	—	P	S	—	—	—
XBB.1.16	Apr 15, 2023	H	T	I	—	—	—	—	—	P	S	L	K	R	P	S	—	—	—
XBB.1.16.1	May 27, 2023	H	T	I	—	—	—	—	—	P	S	L	K	R	P	S	—	—	—
XBB.2.3	May 6, 2023	H	T	I	—	—	—	—	—	P	S	L	K	—	P	S	—	—	S

**Abbreviations:** BA = B.1.1.529; CDT = COVID Data Tracker; RBD = receptor binding domain.

* Amino acid substitutions in the receptor binding domain (relative to a BA.4/BA.5 spike protein reference sequence) were included if they were present in ≥50% of sequences belonging to a given Pango lineage. The BA.4/BA.5 spike protein was used as a reference because of its inclusion in the bivalent mRNA COVID-19 booster vaccines. Residues conserved or with substitutions present in <50% of a lineage are not shown.

^†^ Lineages reaching a prevalence of ≥1% with spike protein substitutions of potential therapeutic relevance and listed separately on the CDT website. https://covid.cdc.gov/covid-data-tracker/#variant-proportions

^§^ Indicates sites of independent substitutions in at least two different evolutionary lineages.

^¶^ Indicates sites identified in a previous study (https://doi.org/10.1038/s41586-021-04385-3) associated with in vitro reductions in binding by monoclonal antibodies that were previously authorized by the Food and Drug Administration.

** Dashes indicate no change from the BA.4/BA.5 reference sequence.

In late fall 2022, the XBB lineage (recombinant of two BA.2 descendant lineages, BM.1.1.1 and BJ.1, with R346T, G446S, N460K, and F486S RBD substitutions) emerged in the United States, reaching <5% prevalence nationally. XBB.1.5, an XBB descendant (harboring an additional S486P substitution) was initially reported in New York City in October 2022 (*5*) and first reached predominance in HHS Region 2 (New York, New Jersey, Puerto Rico, and the U.S. Virgin Islands) on December 31, 2022, and Region 1 (Connecticut, Maine, Massachusetts, New Hampshire, Rhode Island, and Vermont) on January 7, 2023 (Figure 2). XBB.1.5 further spread south and west to attain national predominance by January 28, 2023, reaching a peak prevalence of 84.1% (95% CI = 81.4%–86.5%) by April 1 (Figure 1).

**FIGURE 2:**
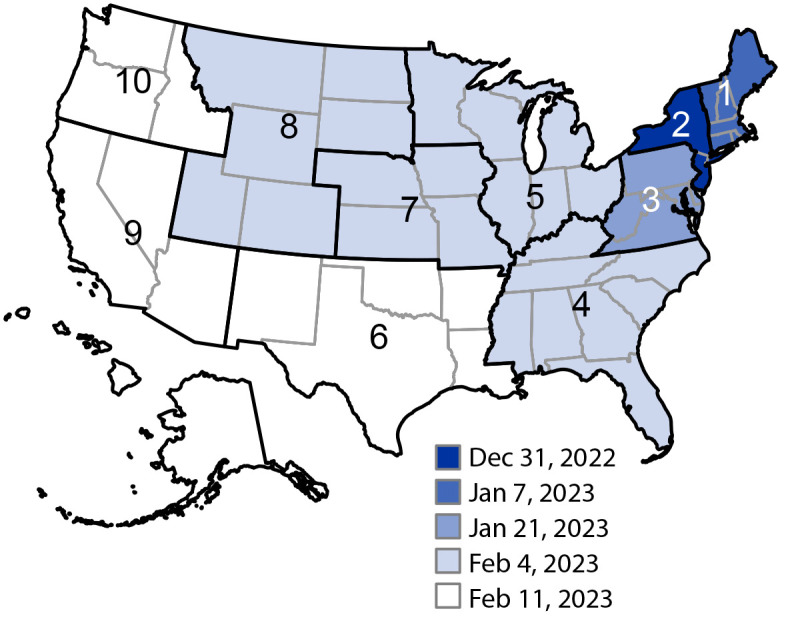
Omicron XBB.1.5 predominance,* by U.S. Department of Health and Human Services Region^†^ and week that the variant became predominant — United States, December 25, 2022–February 11, 2023 **Abbreviation**: HHS = U.S. Department of Health and Human Services. * The timing of XBB.1.5 predominance was defined as the week ending date during which the variant proportion estimate exceeded 50% in each HHS region. https://covid.cdc.gov/covid-data-tracker/#variant-proportions ^†^ HHS Region 2 includes data from Puerto Rico and the U.S. Virgin Islands. HHS Region 3 includes data from the District of Columbia. HHS Region 9 includes data from American Samoa, Guam, and the Northern Mariana Islands. https://www.hhs.gov/about/agencies/iea/regional-offices/index.html

As of May 13, 2023, the commonly circulating Omicron lineages were XBB.1.5 (61.5%; 95% CI = 56.4%–66.4%), XBB.1.9.1 (10.0%; 95% CI = 6.8%–14.1%), and XBB.1.16 (9.4%; 95% CI = 6.9%–12.5%), with approximately a 19% combined prevalence of other circulating lineages, including XBB (5.3%), XBB.1.9.2 (4.5%), XBB.2.3 (3.2%), XBB.1.16.1 (2.4%), and XBB.1.5.1 (1.9%). Whereas many circulating XBB lineages share the XBB.1.5 spike sequence, XBB.1.16 and XBB.1.16.1 also contain the K478R RBD substitution and XBB.2.3 also contains the P521S substitution (Table). During the week ending May 13, 2023, the fastest doubling times were observed for XBB.1.16 (15.7 days; 95% CI = 13.9–17.9), XBB.1.16.1 (16.7 days; 95% CI = 14.3–20.2), and XBB.2.3 (20.3 days; 95% CI = 16.6–26.0).

The fastest doubling times among lineages assessed at 1% prevalence during January 2, 2022–May 13, 2023, occurred for BA.2.12.1 (5.4 days; 95% CI = 4.8–6.1), BQ.1.1 (6.3 days; 95% CI = 5.5–7.2), BA.5 (6.8 days; 95% CI = 5.9–8.2), and XBB.1.5 (7.0 days; 95% CI = 5.8–8.6). In comparison, the doubling time for Omicron B.1.1.529 was 3.2 days (*1*). BA.5, XBB.1.5, and BA.1.1 remained predominant for the longest durations (19, 16, and 10 weeks, respectively). The number of cases attributed to each lineage was highest for BA.1.1 (14 million), B.1.1.529 (9.8 million) and BA.5 (8.0 million) (Figure 1) (Supplementary Figure 2, https://stacks.cdc.gov/view/cdc/129516). As of May 13, 2023, XBB.1.5 was associated with 1.8 million cases, with numbers expected to continue increasing.

Beginning May 13, 2023, after the expiration of the public health emergency declaration (*4*) and in response to declining numbers of cases and sequenced specimens, methodologic changes were made regarding the analysis of SARS-CoV-2 genomic surveillance data. The reporting cadence and unit of analysis changed from weekly to biweekly, with variant proportions estimated for 2-week periods and nowcast predictions conducted for the most recent 4 weeks,^§§§§^ and state-specific estimates were discontinued. For calculating survey weights, the level and source for information on positive test results changed to regional-level data from the National Respiratory and Enteric Virus Surveillance System (NREVSS)^¶¶¶¶^ (*6*). The previous and updated analytic methods using CELR- and NREVSS-derived survey weights, respectively, produced similar variant proportion estimates for all lineages. An example comparison of national and regional proportions of XBB.1.5 demonstrates the consistency between methodologies (Supplementary Figure 3, https://stacks.cdc.gov/view/cdc/129517).

## Discussion

During January 2022–May 2023, CDC’s genomic surveillance system detected the emergence and changing prevalence of multiple Omicron lineages nationwide. Predominant lineages included BA.1.1, BA.2, and BA.2.12.1 in the first half of 2022 and BA.5 and BQ.1/BQ.1.1 (combined) in the second half. Surges in COVID-19 cases were associated with the emergences of these variants. The rise of XBB.1.5 to predominance in 2023 was characterized by an expansion from the northeastern United States to southeastern and western regions. Multiple Omicron lineages independently acquired similar substitutions (e.g., R346T, K444T, N460K, and F486S/P) in the spike RBD, suggesting that these sites are under selective pressure in the population and drive enhanced viral circulation (*7*). Accordingly, these substitutions have been observed to be associated with escape from neutralizing antibodies, including previously authorized monoclonal antibody therapies (*7*,*8*), and the S486P substitution observed in some XBB-descendent lineages also has been observed to increase infectivity via enhanced angiotensin-converting enzyme 2 receptor binding affinity (*9*). XBB lineages with additional substitutions compared with XBB.1.5, namely XBB.1.16, XBB.1.16.1, and XBB.2.3, had the fastest doubling times as of May 13, 2023.

Data on SARS-CoV-2 Omicron variant proportions helped guide decisions to revoke the emergency use authorizations for different monoclonal antibody therapies with decreased clinical efficacy against various Omicron lineages starting winter 2021.***** Data on variant proportions were also used by the Food and Drug Administration (FDA) to recommend the inclusion of BA.4/BA.5 in updated (bivalent) vaccines in June 2022 and are expected to guide decisions about the composition of future COVID-19 vaccines.^†††††^

The findings in this report are subject to at least four limitations. First, early SARS-CoV-2 variant proportion estimates might have low precision because of relatively limited data availability and biases in the timing of specimen collection or sequence submission. These effects can be exacerbated by sequencing and reporting lag time (e.g., holidays) or laboratory issues, such as lineage-specific sequencing failures. Second, continued decreases in the number of sequencing specimens available over time affect precision; for this reason, state-specific estimates were discontinued in May 2023. Third, current analyses might differ from previous analyses because of fluctuations in sequencing data sources, changes in Pango lineage definitions, and methodologic updates. Finally, estimates of COVID-19 cases attributed to more recent lineages are affected by case underascertainment because of increasing at-home test use and other changes in test-seeking behaviors.

CDC has maintained national SARS-CoV-2 genomic surveillance since December 2020 to monitor variant proportions and aid in making timely decisions on prevention strategies, including vaccines and therapeutics. Analytic methods have been updated to maintain robust and representative estimates as the availability of sequencing specimens has declined; it is reassuring that the previous and updated weighting methodologies produced consistent estimates. Continued monitoring of SARS-CoV-2 variants in the U.S. population is key for guiding public health action, including FDA authorizations for COVID-19 therapeutics and strain selection for vaccines.

**Summary**

**What is already known about this topic?**

CDC has used genomic surveillance to monitor trends in circulating U.S. SARS-CoV-2 variants since December 2020, including the emergence of the Omicron variant at the end of 2021.

**What is added by this report?**

Weekly estimates of variant proportions during January 2, 2022–May 13, 2023, identified the emergence and subsequent predominance of multiple Omicron lineages in the United States, including BA.2, BA.2.12.1, BA.5, and XBB.1.5. Repeated independent substitutions in the spike protein suggested convergent evolution related to immune evasion. Analytic methods for variant proportion estimation have been updated as numbers of cases and sequenced specimens have declined.

**What are implications for public health practice?**

Ongoing genomic surveillance can identify emerging SARS-CoV-2 variants and guide vaccine and therapeutic development and use.

## Footnotes

* These authors contributed equally to this report.

†
https://www.cdc.gov/coronavirus/2019-ncov/variants/cdc-role-surveillance.html

§ Sequences from public sequence data repositories are limited to those meeting baseline surveillance criteria, which ensures that they appropriately capture geographic, demographic, and clinical diversity. https://www.aphl.org/programs/preparedness/Crisis-Management/Documents/Technical-Assistance-for-Categorizing-Baseline-Surveillance-Update-Oct2021.pdf

¶ https://covid.cdc.gov/covid-data-tracker/#variant-proportions; https://data.cdc.gov/Laboratory-Surveillance/SARS-CoV-2-Variant-Proportions/jr58-6ysp

** A consensus sequence is produced by aligning SARS-CoV-2 nucleotide sequences generated through sequencing a sample and then determining the most common nucleotide at each position. A consensus sequence is an interoperable genomic surveillance unit that can be combined from laboratory sources.

†† Quality filters included limiting sequences to include only human-derived sources and U.S-specific sequences and excluding those with invalid state names and laboratory sources.

§§
https://www.hhs.gov/about/agencies/iea/regional-offices/index.html

¶¶ Before August 13, 2022, nowcasts were used to produce estimates for only the most recent 2 weeks.

*** Variant proportion estimation methods account for the complex survey design, with weights based on the weekly estimated number of infections represented by each SARS-CoV-2 sequence; weights are trimmed to the 99th percentile. Each submitting laboratory source was considered a primary sampling unit, and the state and week of sequence sample collection were considered strata. The updated code, weight derivations, and nowcast model equations for the variant proportion estimation methods are available online. https://github.com/CDCgov/SARS-CoV-2_Genomic_Surveillance

††† Beginning October 11, 2022, growth rate and nowcast estimates were conducted for any lineages accounting for ≥0.5% of sequences nationwide (unweighted) in the last week before nowcast estimates. Lineages with a prevalence <1% or without spike protein substitutions of potential therapeutic or clinical relevance were aggregated with their parental lineage in the final estimates.

§§§ Doubling times for proportions of specific lineages are based on instantaneous growth rates from the multinomial nowcasting model. Doubling times were assessed either 1) when a lineage reached 1% prevalence, for comparisons of doubling times across all lineages, or 2) during the most recent week of data availability, to assess growth trajectories for currently circulating lineages.

¶¶¶ 45 C.F.R. part 46.102(l)(2), 21 C.F.R. part 56; 42 U.S.C. Sect.241(d); 5 U.S.C.0 Sect.552a; 44 U.S.C. Sect. 3501 et seq.

**** SARS-CoV-2 sequences originated from the 50 U.S. states, the District of Columbia, American Samoa, Guam, Northern Mariana Islands, Puerto Rico, and U.S. Virgin Islands.

†††† An unscaled dendrogram depicting the phylogenetic relationships between Omicron lineages is available on CDC’s COVID Data Tracker. https://covid.cdc.gov/covid-data-tracker/#variant-summary

§§§§ Beginning May 11, 2023, weighted variant proportions were estimated for the six 2-week periods (12 weeks total) before the two most recent 2-week periods for select lineages accounting for ≥1% (unweighted) of sequences nationwide. Nowcast predictions were used to produce estimates for the two most recent 2-week periods. Nowcasts were also conducted for lineages accounting for ≥0.5% (unweighted) of sequences nationwide during the first 2-week nowcast period to improve accuracy by accounting for differential growth rates of grouped sublineages.

¶¶¶¶ Test positivity data (weekly numbers of positive specimens and total tests administered) from CELR were no longer available after the expiration of the public health emergency declaration (https://healthdata.gov/dataset/COVID-19-Diagnostic-Laboratory-Testing-PCR-Testing/j8mb-icvb). Beginning May 11, 2023, the percentage of positive nucleic acid amplification test results by HHS Region from NREVSS (https://www.cdc.gov/surveillance/nrevss/index.html), which correlate well with CELR data (https://doi.org/10.15585/mmwr.mm7219e2), were used with the number of positive specimens by state from CELR to estimate survey design weights.

***** https://www.fda.gov/drugs/emergency-preparedness-drugs/emergency-use-authorizations-drugs-and-non-vaccine-biological-products; https://www.covid19treatmentguidelines.nih.gov/tables/variants-and-susceptibility-to-mabs/

†††††
https://www.who.int/news/item/18-05-2023-statement-on-the-antigen-composition-of-covid-19-vaccines
